# Major Bloodstream Infection-Causing Bacterial Pathogens and Their Antimicrobial Resistance in South Korea, 2017–2019: Phase I Report From Kor-GLASS

**DOI:** 10.3389/fmicb.2021.799084

**Published:** 2022-01-06

**Authors:** Dokyun Kim, Eun-Jeong Yoon, Jun Sung Hong, Min Hyuk Choi, Hyun Soo Kim, Young Ree Kim, Young Ah Kim, Young Uh, Kyeong Seob Shin, Jeong Hwan Shin, Jeong Su Park, Kyoung Un Park, Eun Jeong Won, Soo Hyun Kim, Jong Hee Shin, Jung Wook Kim, SungYoung Lee, Seok Hoon Jeong

**Affiliations:** ^1^Department of Laboratory Medicine and Research Institute of Bacterial Resistance, Yonsei University College of Medicine, Seoul, South Korea; ^2^National Institute of Health, Korea Disease Control and Prevention Agency, Cheongju, South Korea; ^3^Department of Laboratory Medicine, Hallym University Dongtan Sacred Heart Hospital, Hallym University College of Medicine, Hwaseong, South Korea; ^4^Department of Laboratory Medicine, Jeju National University School of Medicine, Jeju, South Korea; ^5^Department of Laboratory Medicine, National Health Insurance Service Ilsan Hospital, Goyang, South Korea; ^6^Department of Laboratory Medicine, Yonsei University Wonju College of Medicine, Wonju, South Korea; ^7^Department of Laboratory Medicine, College of Medicine, Chungbuk National University, Cheongju, South Korea; ^8^Department of Laboratory Medicine and Paik Institute for Clinical Research, Inje University College of Medicine, Busan, South Korea; ^9^Department of Laboratory Medicine, Seoul National University College of Medicine, Seoul National University Bundang Hospital, Seongnam, South Korea; ^10^Department of Laboratory Medicine, Chonnam National University Medical School, Gwangju, South Korea

**Keywords:** antimicrobial resistance, Kor-GLASS, WHO, GLASS, surveillance

## Abstract

To monitor national antimicrobial resistance (AMR), the Korea Global AMR Surveillance System (Kor-GLASS) was established. This study analyzed bloodstream infection (BSI) cases from Kor-GLASS phase I from January 2017 to December 2019. Nine non-duplicated Kor-GLASS target pathogens, including *Staphylococcus aureus*, *Enterococcus faecalis*, *Enterococcus faecium*, *Streptococcus pneumoniae*, *Escherichia coli*, *Klebsiella pneumoniae*, *Pseudomonas aeruginosa*, *Acinetobacter* spp., and *Salmonella* spp., were isolated from blood specimens from eight sentinel hospitals. Antimicrobial susceptibility testing, AMR genotyping, and strain typing were carried out. Among the 20,041 BSI cases, 15,171 cases were caused by one of the target pathogens, and 12,578 blood isolates were collected for the study. Half (1,059/2,134) of *S. aureus* isolates were resistant to cefoxitin, and 38.1% (333/873) of *E. faecium* isolates were resistant to vancomycin. Beta-lactamase-non-producing ampicillin-resistant and penicillin-resistant *E. faecalis* isolates by disk diffusion method were identified, but the isolates were confirmed as ampicillin-susceptible by broth microdilution method. Among *E. coli*, an increasing number of isolates carried the *bla*_CTX–M–27_ gene, and the ertapenem resistance in 1.4% (30/2,110) of *K. pneumoniae* isolates was mostly (23/30) conferred by *K. pneumoniae* carbapenemases. A quarter (108/488) of *P. aeruginosa* isolates were resistant to meropenem, and 30.5% (33/108) of those carried acquired carbapenemase genes. Over 90% (542/599) of *A. baumannii* isolates were imipenem-resistant, and all except one harbored the *bla*_OXA–23_ gene. Kor-GLASS provided comprehensive AMR surveillance data, and the defined molecular mechanisms of resistance helped us to better understand AMR epidemiology. Comparative analysis with other GLASS-enrolled countries is possible owing to the harmonized system provided by GLASS.

## Introduction

The World Health Organization (WHO) has declared that antimicrobial resistance (AMR) is one of the top ten global public health threats, and as a core element of the global action plan to control this menace, the Global AMR Surveillance System (GLASS) was launched in 2015 (World Health Organization [WHO], 2015). The GLASS is a case-finding-based surveillance system that collates AMR data of major pathogens with clinical data of patients with the infection (World Health Organization [WHO], 2016). As the GLASS manual standardized AMR surveillance research, global AMR monitoring became available for infection-causing bacterial pathogens under a harmonized method carried out by GLASS-enrolled countries (World Health Organization [WHO], 2015). In line with the efforts of the WHO, the Korea Disease Control and Prevention Agency (former Korea Centers for Disease Control and Prevention) established an AMR surveillance system compatible with the GLASS in 2016, which is running under the name Kor-GLASS (Lee et al., 2018a).

In addition to the case-finding setup of the GLASS, Kor-GLASS collected all target bacterial isolates (Lee et al., 2018a). The isolates of each target species were collected in a collection center and transferred to specialized analysis centers. Then, the bacterial species of each isolate were identified, and antimicrobial susceptibility testing was conducted. Following the basic characterization of the collected isolates, characterization for AMR genotyping and strain typing was carried out, and finally, the isolates were kept in a long-term storage system. Kor-GLASS has a good reputation as a desirable national AMR surveillance system due to its solid structure, superb performance, and completion of GLASS reports (Lee et al., 2018b; Simonsen, 2018). After the successful pilot phase of Kor-GLASS with six sentinel hospitals in 2016, the system was advanced to phase I (2017–2019) through (i) including two more sentinel hospitals for two additional districts, (ii) establishing an independent quality assessment center, and (iii) constructing a web-based data management system.

Bloodstream infection (BSI) is a serious infectious disease associated with high morbidity and mortality. Globally, the common bacterial pathogens that cause BSIs are *Escherichia coli* and *Staphylococcus aureus* (Diekema et al., 2019), with a few regional variations caused either by demographic traits, such as the population, geographic location, socioeconomic status, medical environment, or by endemic AMR in clinical pathogens (Huggan et al., 2010; Laupland, 2013; Buetti et al., 2017; Diekema et al., 2019; Pfaller et al., 2020). Additionally, the AMR in BSI-causative pathogens varies by country under the influence of antimicrobial usage, health care infrastructure including the accessibility of medical services and health care workers, and the central policy for infection prevention and control. The proportions of methicillin-resistant *S. aureus* (MRSA) were very different between countries, even on the European continent, 37.6% in Greece and 1.8% in Sweden (European Centre for Disease Prevention and Control [ECDC], 2020), and different rates of resistance to carbapenems in *Acinetobacter baumannii* were found in two countries in the Far East, very high in South Korea (90%) and very low in Japan (5%) (Japan Nosocomial Infecions Surveillance [JANIS], 2021).

Here, we present the data of BSI cases from the phase I Kor-GLASS from January 2017 to December 2019 regarding the incidence of BSIs, BSI-causing pathogens, and their AMR. The global AMR data obtained from the harmonized GLASS method (World Health Organization [WHO], 2019) enabled comparative analysis of AMR in South Korea with findings from other parts of the world.

## Materials and Methods

### Collection of Target Blood Isolates and Clinical Data of Patients From Sentinel Hospitals

Between January 2017 and December 2019, non-duplicated blood isolates of *S. aureus*, *Enterococcus faecalis*, *Enterococcus faecium*, *Streptococcus pneumoniae*, *E. coli*, *Klebsiella pneumoniae*, *Pseudomonas aeruginosa*, *Acinetobacter* spp., and *Salmonella* spp. obtained from patients in eight sentinel hospitals in South Korea were prospectively collected (Supplementary Figure 1). Demographic information, including the age and sex of each patient, was investigated. The type of admission was classified into three categories: outpatient department (OPD), general ward (GW), and intensive care unit (ICU). The type of infection was categorized into hospital-originated (HO) and community-originated (CO) infections through the criteria of hospitalization days before taking the blood specimen, defining ≥2 days as HO and lower values as CO. The collected isolates were transferred to the analysis centers twice a month, and clinical information was uploaded to a web-based database. In addition, the number of patients subjected to blood culture, number of patients with positive blood culture, frequency of BSI occurrence by causative bacterial or fungal species including both target and non-target pathogens of Kor-GLASS, and patient-days of inpatients in each hospital were retrospectively retrieved through electronic medical records.

### Ethics Statement

In this study, clinical data of the patients including sex, age, type of admission, and type of infection were investigated by retrospective chart review with personally identifiable information being removed. Due to the purely observational nature and very low risk to individual privacy of the participants, this study was approved by all local institutional review boards of the eight sentinel hospitals and exempted from the requirement of informed consent.

### Microbiological Characterization in Analysis Centers

Bacterial identification of eight Kor-GLASS target pathogens were performed by matrix-assisted laser desorption ionization–time of flight mass spectrometry (MALDI Biotyper, Bruker Daltonics, Bremen, Germany), and confirmed by 16S rRNA sequencing (*S. aureus*, enterococci, *E. coli*, *K. pneumoniae*, *Salmonella* spp., and *P. aeruginosa*), *rpoB* sequencing (*Acinetobacter* spp.), or *recA, tuf*, and *sodA* sequencing (*S. pneumoniae*) in the analysis centers. AST was performed by the disk diffusion method, and the supplemental broth microdilution method was carried out for the selected drugs. The lists of antimicrobial agents and quality control strains tested in this study are presented in Supplementary Table 1. Both ASTs followed the Clinical and Laboratory Standards Institute or European Committee on Antimicrobial Susceptibility Testing (EUCAST) guidelines (Clinical and Laboratory Standards Institute [CLSI], 2016; European Committee on Antimicrobial Susceptibility Testing [EUCAST], 2016).

### Antimicrobial Resistance Genotyping

Antimicrobial resistance genotyping and strain typing were performed following the Kor-GLASS manual (Korea Disease Control and Prevention Agency [KDCA], 2017). Cefoxitin-resistant *S. aureus* isolates were subjected to PCR for the detection of the *mecA* and *mecC* genes, and the type of the staphylococcal cassette chromosome *mec* (SCC*mec*) was determined by multiplex PCR assays (Oliveira and de Lencastre, 2002). The genes for staphylococcal virulence factors toxic shock syndrome toxin-1 (*tst-1*) and Panton-Valentine-Leucocidin (*pvl*) were assessed by PCR (Kim et al., 2019a). For strain typing, PCR and sequencing of the staphylococcal protein A gene repeat region were performed to determine the *spa* type of the isolates by comparison to the database of the RidomSpaServer website (Koreen et al., 2004). Penicillin- and/or ampicillin-resistant enterococcal isolates were subjected to a cefinase disk test (BD Biosciences, Sparks, MD, United States) for the production of β-lactamase. Enterococcal isolates that were intermediate or resistant to vancomycin and/or teicoplanin were subjected to PCR to detect the *vanA*, *vanB*, and *vanM* genes (Elsayed et al., 2001; Teo et al., 2011). Gram-negative bacilli exhibiting resistance phenotypes of extended-spectrum β-lactamases (ESBLs), AmpC β-lactamases, and carbapenemases according to the EUCAST criteria were subjected to PCR and sequencing for the corresponding genes encoding CTX-M-type ESBLs (CTX-M-1, CTX-M-2, CTX-M-9, and CTX-M-25 types), plasmid-mediated AmpC β-lactamases (CMY-1 and CMY-2 types, DHA, FOX, ACC, and ACT), and carbapenemases [two serine carbapenemases, *K. pneumoniae* carbapenemase (KPC) and Guiana extended-spectrum β-lactamase (GES), and three metallo-β-lactamases (MBLs), New Delhi MBL (NDM), imipenemase (IMP), and Verona integron-encoded MBL (VIM)] (European Committee on Antimicrobial Susceptibility Testing [EUCAST], 2017). The information of the primers used in this study is summarized in Supplementary Table 2.

### External Quality Control

For external quality control of the bacterial species identification and AST, 5% of the analysis centers’ testing results were evaluated by comparison with those conducted in an independent quality control center. In addition, the analysis centers were regularly certified by the external quality assurance program of the quality control center every 3 months.

## Results

### Case-Finding for Bloodstream Infections

**FIGURE 1:**
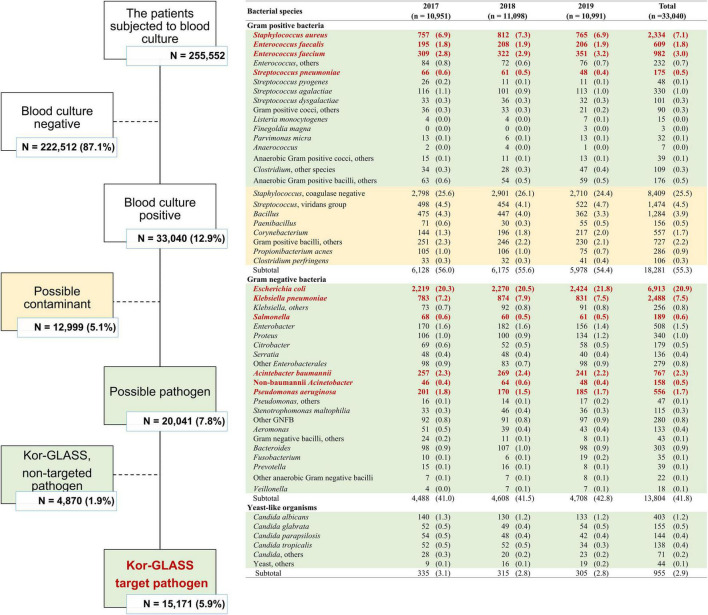
Bloodstream infection cases stratified according to the pathogen during the study period.

In the 3-year study period, a total of 255,552 patients in eight sentinel hospitals were subjected to blood culture, and 33,040 (12.9%) blood cultures were positive for at least one bacterial or fungal pathogen (Figure 1). Excluding 12,999 (39.3%) probable contaminated blood cultures (Horan et al., 2008), a total of 20,041 BSI cases occurred during the study period. Approximately one-quarter (26.4%, 5,282/20,041) of blood cultures were positive for Gram-positive bacteria, including *S. aureus* (*n* = 2,334), *E. faecium* (*n* = 982), *E. faecalis* (*n* = 609), and *S. pneumoniae* (*n* = 175). Two-thirds (68.9%, 13,804/20,041) of the blood cultures were positive for Gram-negative bacteria, i.e., Enterobacterales (*n* = 11,288), including *E. coli* (*n* = 6,913), *K. pneumoniae* (*n* = 2,488), *Salmonella* spp. (*n* = 189), and glucose-non-fermenting Gram-negative bacilli (*n* = 1,923), including *A. baumannii* (*n* = 767) and *P. aeruginosa* (*n* = 556). As minor cases, 783 (3.9%) blood cultures were positive for anaerobes, and 955 (4.8%) blood cultures were positive for yeast-like organisms.

In total, 15,171 BSI cases were caused by nine Kor-GLASS target pathogens, and among those, a total of 12,578 blood isolates were included in this study for further evaluation, of which 2,594 (17.1%) blood isolates were lost due to human error, either by handling errors (*n* = 2,256) or by microbial cross-contamination (*n* = 337).

### Kor-GLASS Target Pathogens Causing Bloodstream Infections

**FIGURE 2:**
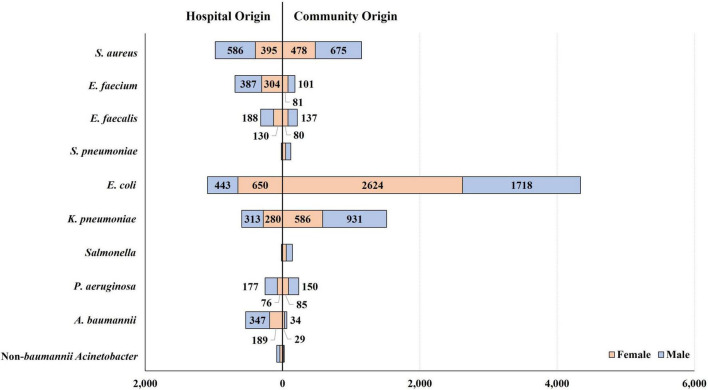
Occurrence of bloodstream infections by patient group, categorized by patient sex and type of infection. *E. coli-*caused BSIs occurred 1.5-fold more frequently in female patients than in male patients, in contrast to the other bacterial BSIs: in terms of the relative incidence of BSI in females to that in males, 0.7 were caused by *S. aureus*, 0.8 by *E. faecium*, 0.7 by *E. faecalis*, 0.7 by *K. pneumoniae*, 0.5 by *P. aeruginosa*, and 0.6 by *A. baumannii*. By type of infection, most *Salmonella* (91.1%, 144/158) and *S. pneumoniae* (87.6%, 120/137) isolates were recovered from patients with CO infection, and *E. coli* was isolated from CO infection four times more frequently than from HO infection. In contrast, *A. baumannii* and *E. faecium* presented the opposite relative frequency of HO infection, as 8.5 and 3.8 times more were isolated from patients with HO infection than those with CO infection, respectively. *S. aureus* was evenly isolated from patients with CO and HO infections.

*Escherichia coli* was the most common BSI-associated species (*n* = 5,435), followed by *S. aureus* (*n* = 2,134), *K. pneumoniae* (*n* = 2,110), *E. faecium* (*n* = 873), *Acinetobacter* spp. (*n* = 607), *E. faecalis* (*n* = 535), *P. aeruginosa* (*n* = 488), *Salmonella* spp. (*n* = 158), and *S. pneumoniae* (*n* = 137). The BSI occurrence varied by patient group, categorized by sex, type of infection, and infection-causing bacterial species (Figure 2).

**FIGURE 3:**
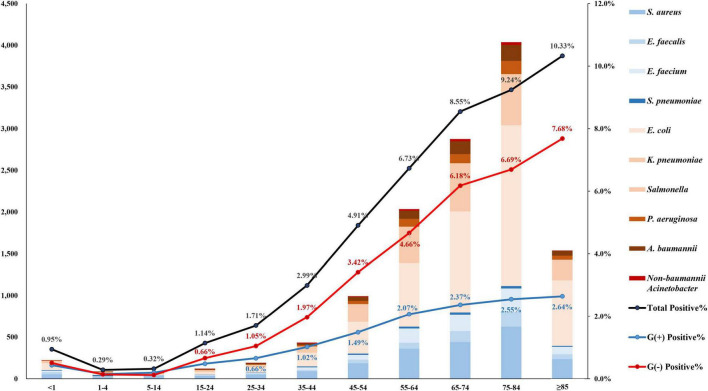
Number of patients with bloodstream infection by target pathogen. The rate of blood culture positivity for target pathogens was the lowest at 0.29% in the age group 1–4, and it increased as the patients became older, up to 10.33% in the age group ≥85. The bar represents the number of patients by pathogen. The black line is the percentage of patients positive for target pathogen, the blue line is that for gram-positive pathogens, and the red line is that for gram-negative pathogens.

The rate of blood culture positivity for target pathogens was the lowest at 0.29% in the age group 1–4, and it increased as the patients became older, up to 10.33% in the age group ≥85 (Figure 3). It was noteworthy that the rate of Gram-negative pathogen-positive blood cultures increased more steeply from 0.12% in the age group 5–14 to 7.68% (64.0 times) in the age group ≥85, while the positive rate of Gram-positive pathogens increased less steeply increased from 0.15% in the age group 1–4 to 2.64% (17.6 times) in the age group ≥85. By admission type, while the *Salmonella* spp. were more frequently isolated from outpatients (55.7%, 88/158) than from inpatients, most (93.0%, 557/599) of the *A. baumannii* BSI cases were identified in inpatients, especially in ICU patients (68.3%, 409/599). *E. coli* was evenly isolated from both outpatients (49.0%, 2,664/5,435) and inpatients (51.0%, 2,771/5,435) (Supplementary Figure 2).

The 3-year total of 6,242,798 patient-days of the eight sentinel hospitals included 5,608,067 patient-days in GWs and 634,731 patient-days in ICUs. In GWs, the incidence of *E. coli* BSI was the most frequent at 40.8 cases/100,000 patient-days, followed by *S. aureus* (16.7 cases/100,000 patient-days) and *K. pneumoniae* (16.0 cases/100,000 patient-days) BSIs; however, in ICUs, *S. aureus* BSI was the most common (84.4 cases/100,000 patients-days), followed by *E. coli* (75.8 cases/100,000 patient-days) and *A. baumannii* (64.4 cases/100,000 patient-days) BSIs (Supplementary Figure 3).

### Antimicrobial Resistance and the Mechanisms of Antimicrobial Resistance in Bloodstream Infection-Causing Pathogens

#### Staphylococcus aureus

**FIGURE 4:**
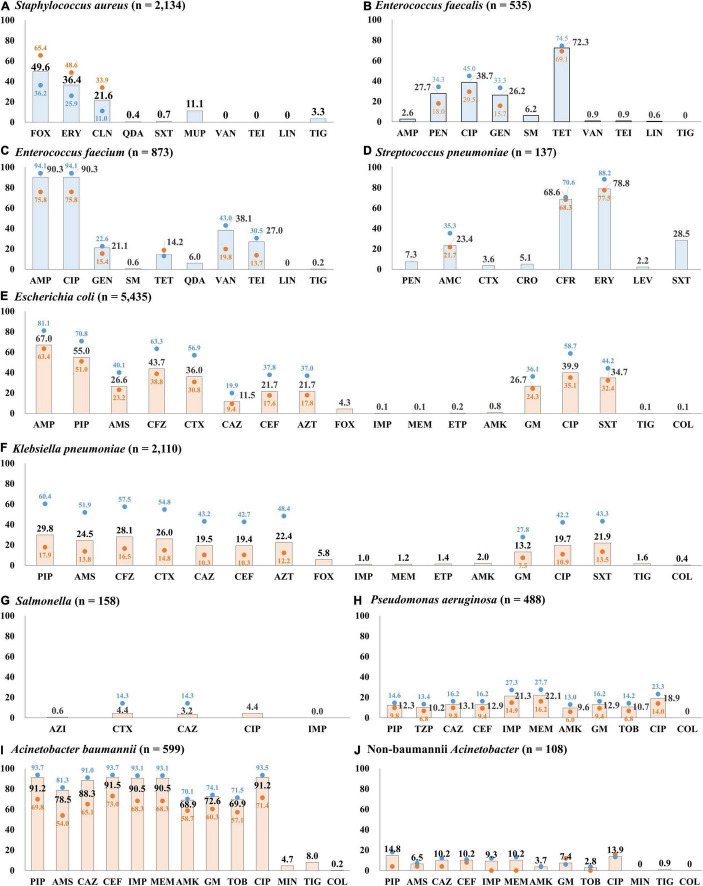
Percentage of resistance to major antimicrobials by infection origin. **(A)** Antimicrobial resistance rates of *S. aureus* blood isolates. **(B)** Antimicrobial resistance rates of *E. faecalis* blood isolates. **(C)** Antimicrobial resistance rates of *E. faecium* blood isolates. **(D)** Antimicrobial resistance rates of *S. pneumoniae* blood isolates. **(E)** Antimicrobial resistance rates of *E. coli* blood isolates. **(F)** Antimicrobial resistance rates of *K. pneumoniae* blood isolates. **(G)** Antimicrobial resistance rates of *Salmonella* blood isolates. **(H)** Antimicrobial resistance rates of *P. aeruginosa* blood isolates. **(I)** Antimicrobial resistance rates of *A. baumannii* blood isolates. **(J)** Antimicrobial resistance rates of non-baumannii *Acinetobacter* blood isolates. The bars indicate the resistance rates of total blood isolates, the blue dots indicate those of isolates from hospital-originated infection, and the red dots indicate those of isolates from community-originated infection. Abbreviations: AMC, amoxicillin-clavulanate; AMK, amikacin; AMP, ampicillin; AMS, ampicillin-sulbactam; AZI, azithromycin; AZT, aztreonam; CAZ, ceftazidime; CEF, cefepime; CFR, cefuroxime; CFZ, cefazolin; CIP, ciprofloxacin; CLN, clindamycin; COL, colistin; CRO, ceftriaxone; CTX, cefotaxime; ERY, erythromycin; ETP, ertapenem; FOX, cefoxitin; GEN, gentamicin; IMP, imipenem; LEV, levofloxacin; LIN, linezolid; MEM, meropenem; MIN, minocycline; MUP, mupirocin; PEN, penicillin; PIP, piperacillin; QDA, quinupristin-dalfopristin; SM, streptomycin; SXT, trimethoprim-sulfamethoxazole; TEI, teicoplanin; TET, tetracycline; TIG, tigecycline; TOB, tobramycin; TZP, piperacillin-tazobactam; VAN, vancomycin.

**FIGURE 5:**
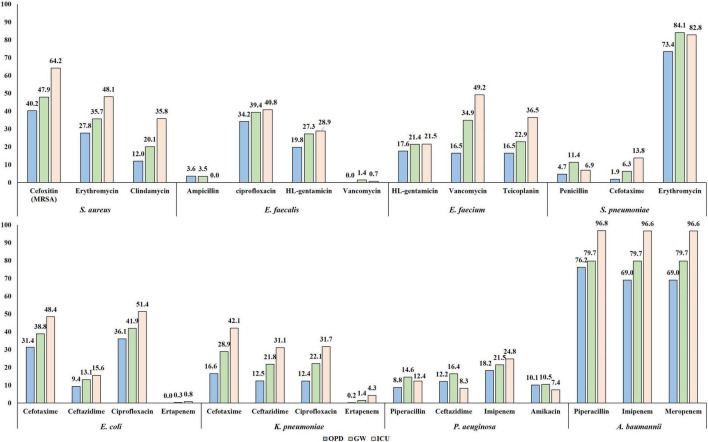
The percentage of resistance to major antimicrobials of bloodstream infection-causing pathogens by admission type. The resistance rates determined by disk diffusion method are presented.

Half (49.6%, 1,059/2,134) of the *S. aureus* blood isolates exhibited resistance to cefoxitin, indicating methicillin-resistant *S. aureus* (MRSA) (Figure 4A). The resistance rates to erythromycin and clindamycin were 36.4% (776/2,134) and 21.6% (460/2,134), respectively. All *S. aureus* isolates were susceptible to anti-MRSA antibiotics, including vancomycin, teicoplanin, and linezolid. MRSA was more frequently isolated from patients with HO infection (65.4%, 642/981) than from those with CO infection (36.2%, 417/1,153). By admission type, *S. aureus* isolates from inpatients in ICUs (64.2%, 344/536) showed a higher rate of resistance to cefoxitin than those from inpatients in GWs (47.9%, 450/939) and from outpatients of OPDs (40.2%, 265/659) (Figure 5).

All the MRSA isolates harbored the *mecA* gene, but none of them harbored the *mecC* gene. Most MRSA isolates carried either SCC*mec* type II (47.5%, 503/1,059) or type IV (50.1%, 531/1,059). Ten (1.0%) MRSA isolates carried SCC*mec* type V, and the SCC*mec* types of the remaining 12 MRSA isolates were not determined by multiplex PCR assay (Supplementary Figure 4). Three-quarters (75.3%, 379/503) of the SCC*mec* type II-carrying MRSA isolates were recovered from patients with HO infection, and half (53.7%, 285/531) of the SCC*mec* type IV-carrying MRSA isolates were from those with CO infection. The SCC*mec* type II-carrying MRSA isolates exhibited peculiar coresistance to erythromycin (94.4%) and clindamycin (80.3%) compared to the MRSA isolates carrying SCC*mec* type IV, 27.5 and 4.9%, respectively. In addition, more SCC*mec* type II-carrying MRSA isolates (76.5%) than type IV-carrying MRSA isolates (3.8%) harbored the *tst-1* gene, and fewer SCC*mec* type II-carrying MRSA isolates (0.6%) than SCC*mec* type IV-carrying MRSA isolates (14.3%) harbored the *pvl* gene. The *spa* types of the MRSA isolates are presented in Supplementary Table 3. The proportion of SCC*mec* type II-carrying MRSA isolates decreased from 26.3% (186/708) in 2017 to 20.5% (140/683) in 2019, prominently among the patients with HO infection, from 40.3% (145/360) in 2017 to 34.2% (101/295). In contrast, the proportion of SCC*mec* type IV-carrying MRSA was invariable regardless of infection type: 26.4% (187/708) in 2017 and 27.1% (185/683) in 2019. Meanwhile, the isolation of SCC*mec* type IV t008 MRSA harboring *pvl* increased from 3.2% (23/708) in 2017 to 5.1% (35/683) in 2019.

#### Enterococci

**FIGURE 6:**
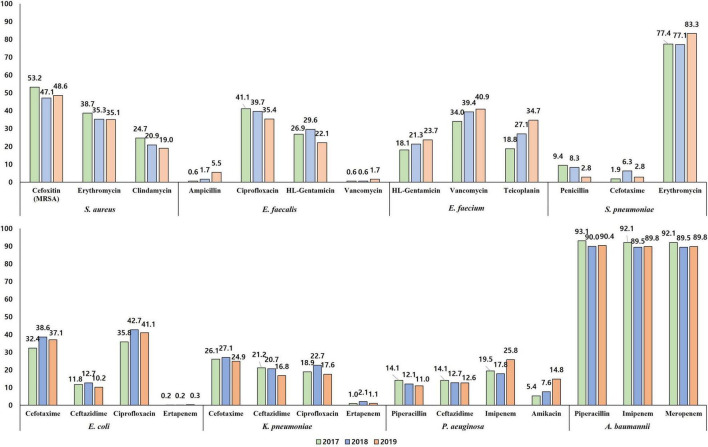
The percentage of resistance to major antimicrobials of bloodstream infection-causing pathogens by year. The resistance rates determined by disk diffusion method are presented.

A quarter (27.9%, 149/535) of *E. faecalis* blood isolates exhibited resistance to penicillin (Figure 4B), and none of them showed positive results in the cefinase disk test. Most (90.6%, 135/149) penicillin-resistant *E. faecalis* isolates exhibited susceptibility to ampicillin, i.e., an ampicillin-susceptible but penicillin-resistant (ASPR) phenotype. The remaining 14 isolates showed resistant phenotype to ampicillin by disk diffusion method, but the minimum inhibitory concentrations (MICs) of ampicillin for the isolates were within susceptible range at 8 μg/mL by broth microdilution method (clinical breakpoint for resistance, ≥16 μg/mL). These beta-lactamase-non-producing ASPR *E. faecalis* isolates exhibiting ampicillin-resistant phenotype by disk diffusion method were recovered from six of eight sentinel hospitals, and the increasing isolation needs attention: 0.6% (1/175) in 2017, 1.7% (3/179) in 2018, and 5.5% (10/181) in 2019 (Figure 6). Furthermore, five (0.9%) vancomycin-resistant *E. faecalis* isolates harboring the *vanA* gene were identified during the study period.

The rate of resistance to vancomycin in *E. faecium* blood isolates was 38.1% (333/873) (Figure 4C). The vancomycin-resistant *E. faecium* (VREfm) isolates were all *vanA*-positive, and *vanB* and *vanM* were not identified in this study. Some (29.1%, 97/333) of the *vanA*-positive VREfm isolates exhibited resistance to vancomycin but intermediate resistance or susceptibility to teicoplanin [MICs of teicoplanin; 4 μg/mL (*n* = 12), 8 μg/mL (*n* = 35), and 16 μg/mL (*n* = 50)], indicating the *vanA* genotype-VanD-like phenotype. VREfm isolates were more frequently recovered from inpatients in ICUs (49.2%, 151/307) than from inpatients in GWs (34.9%, 168/481) and outpatients (16.5%, 14/85) (Supplementary Figure 2). The rate of vancomycin resistance in the *E. faecium* isolates increased from 34.0% in 2017 to 39.4% in 2018 and 40.9% in 2019 (Figure 6).

#### Streptococcus pneumoniae

Although the rate of resistance to penicillin in the *S. pneumoniae* blood isolates was low (7.3%, 10/137), intermediate resistance to penicillin (30.7%, 42/137) was common in *S. pneumoniae*. Among the 52 penicillin-non-susceptible *S. pneumoniae* blood isolates, five (3.6%) isolates exhibited coresistance to cefotaxime (Figure 4D). Three isolates were resistant to levofloxacin (2.2%), while 108 isolates (78.8%) exhibited resistance to erythromycin.

#### Escherichia coli

The rate of resistance to cefotaxime was three times higher (36.0%, 1,959/5,435) than that to ceftazidime (11.5%, 626/5,435) in *E. coli* blood isolates (Figure 4E). Most isolates with the ESBL phenotype harbored at least one *bla*_CTX–M_ gene: 966 isolates harbored the *bla*_CTX–M–1_ group, 810 isolates harbored the *bla*_CTX–M–9_ group, and 67 isolates harbored both the *bla*_CTX–M–1_ group and the *bla*_CTX–M–9_ group (Supplementary Figure 5). No isolates were positive for *bla*_CTX–M–2_ group or *bla*_CTX–M–25_ group. Among the Group 1 CTX-M ESBLs, CTX-M-15 was the most common (74.8% 773/1,033), followed by CTX-M-55 (13.7%, 142/1,033). CTX-M-14 (43.0%, 377/877) was the most common CTX-M-9 group ESBL, followed by CTX-M-27 (38.2%, 335/877), and the *bla*_CTX–M–27_-carrying isolates increased every year: 4.0% (70/1,772) in 2017, 6.0% (104/1,724) in 2018, and 8.3% (161/1,939) in 2019. The rate of resistance to cefoxitin in *E. coli* blood isolates was low (4.3%, 235/5,435), and 139 isolates (59.1%) were positive for either *bla*_DHA–1_ (*n* = 76) or *bla*_CMY–2_ (*n* = 63). Finally, *E. coli* blood isolates resistant to ertapenem (0.2%, 12/5,435) were still rarely observed, and nine of them possessed either *bla*_KPC–2_ (*n* = 3), *bla*_KPC–18_ (*n* = 1), *bla*_NDM–5_ (*n* = 1), or *bla*_OXA–181_ (*n* = 4). The rate of resistance to ciprofloxacin was 39.9% (2,168/5,435).

#### Klebsiella pneumoniae

The rate of resistance to cefotaxime (26.0%, 549/2,110) was higher than that to ceftazidime (19.5%, 411/2,110) in the *K. pneumoniae* blood isolates, but the difference was not as prominent as in the *E. coli* blood isolates (Figure 4F). *K. pneumoniae* isolates exhibiting resistance to extended-spectrum cephalosporins were more frequently isolated from patients with HO infection (54.8 and 43.2% of the isolates were resistant to cefotaxime and to ceftazidime, respectively) than from patients with CO infection (19.5 and 14.8% of the isolates were resistant to cefotaxime and to ceftazidime, respectively). The CTX-M-1 group (78.5%, 431/549), including CTX-M-15 (*n* = 407) and CTX-M-55 (*n* = 5), was the dominant ESBL type in *K. pneumoniae* blood isolates, while the CTX-M-9 group (14.0%, 77/549), including CTX-M-14 (*n* = 60) and CTX-M-27 (*n* = 8), was less frequently identified (Supplementary Figure 5). The resistance rate to cefoxitin was similar (5.8%, 123/2,110) to that of *E. coli* isolates, half (50.4%, 62/123) of cefoxitin-resistant *K. pneumoniae* isolates harbored the *bla*_DHA–1_ gene, and no other genes for plasmid-mediated AmpC β-lactamase were identified. Carbapenem resistance was more frequently identified in *K. pneumoniae* blood isolates than in *E. coli* blood isolates: 1.4% (30/2,110) to ertapenem, 1.2% (*n* = 25) to meropenem, and 1.0% (*n* = 22) to imipenem. Among the 30 ertapenem-resistant *K. pneumoniae* isolates, 23 were positive for *bla*_KPC_ (22 *bla*_KPC–2_-positive isolates and one *bla*_KPC–4_-positive isolate), and no gene for non-KPC carbapenemase was identified in this study.

#### *Salmonella* spp.

Among the 158 *Salmonella* blood isolates, 119 were susceptible to all antimicrobials tested in this study, and only seven (4.4%) isolates were resistant to cefotaxime (Figure 4G). Among those, three cefotaxime-resistant *Salmonella* isolates harbored *bla*_CTX–M–55_, and each harbored either *bla*_CTX–M–15_ or *bla*_CTX–M–65_. Although the rate of resistance to ciprofloxacin was not high, at 4.4% (7/158), *Salmonella* isolates exhibiting intermediate resistance to ciprofloxacin were not rare (20.3%, 32/158).

#### Pseudomonas aeruginosa

Resistance to piperacillin was identified in 12.3% (60/488) of *P. aeruginosa* blood isolates (Figure 4H). The rates of resistance to antipseudomonal cephalosporins were approximately 13% [13.1% (64/488) to ceftazidime and 12.9% (63/488) to cefepime], and those to carbapenems were over 20% [21.3% (104/488) to imipenem and 22.1% (108/488) to meropenem]. Among the 108 meropenem-resistant isolates, one-third (30.5%, 33/108) possessed the acquired carbapenemase genes, including 17 *bla*_IMP–6_-positive isolates, eight *bla*_NDM–1_-positive isolates, seven *bla*_GES_-positive isolates (three for *bla*_GES–9_, three for *bla*_GES–14_, and one for *bla*_GES–5_), and one *bla*_VIM–2_-positive isolate, and most (87.9%, 29/33) of them exhibited coresistance to amikacin. The rate of resistance to amikacin in *P. aeruginosa* isolates was 9.6% (47/488), and the rate of amikacin resistance increased every year: 5.4% (8/149) in 2017, 7.6% (12/157) in 2018, and 14.8% (27/182) in 2019 (Figure 6).

#### *Acinetobacter* spp.

The rates of AMR in *Acinetobacter* blood isolates were distinctively high in *A. baumannii* (Figure 4I). The species showed prominently high rates of resistance to β-lactam antimicrobials, including piperacillin (91.2%, 546/599), ceftazidime (88.3%, 529/599), cefepime (91.5%, 548/599), imipenem (90.5%, 542/599), and meropenem (90.5%, 542/599). Among the 542 imipenem-resistant *A. baumannii* isolates, 541 harbored *bla*_OXA–23_, and one isolate carried IS*Aba1-bla*_OXA–51_. In contrast, non-*baumannii Acinetobacter* isolates exhibited less than 15% resistance rates to all antimicrobials tested in this study, and only ten (9.3%) isolates were resistant to imipenem, including four *bla*_OXA–23_-positive isolates and six MBL gene-harboring isolates (three *bla*_NDM–1_-positive isolates, two *bla*_IMP–1_-positive isolates, and one *bla*_VIM–2_-positive isolate) (Figure 4J). Resistance to minocycline (4.7% 28/599) and tigecycline (8.0% 48/599) was not frequently observed in *A. baumannii* isolates, and only one *A. baumannii* isolate exhibited resistance to colistin.

## Discussion

Among the objectives of GLASS, the outstanding part of the system design is that a comprehensive analysis of comparative AMR data is available for the participating countries out of the regularly reporting AMR burden for selected indicators and AMR data. The BSI incidence caused by Gram-negative organisms was 2.6 times greater than that caused by Gram-positive organisms, and *E. coli* was the most common BSI-causing pathogen in this study. For better comparative analysis of the disease burden, the relative incidences (rIs) of BSIs by causative bacterial species are useful to overcome the biased interpretation caused by the dissimilar scales of the surveillance system and participating hospitals in different GLASS-enrolled countries. Since the predominance of *E. coli* among BSI-causative pathogens is common in most countries around the world (Diekema et al., 2019), the rI of other-bacterial BSI to the incidence of *E. coli*-BSI (rI-Ec) was used for the comparative analysis.

**FIGURE 7:**
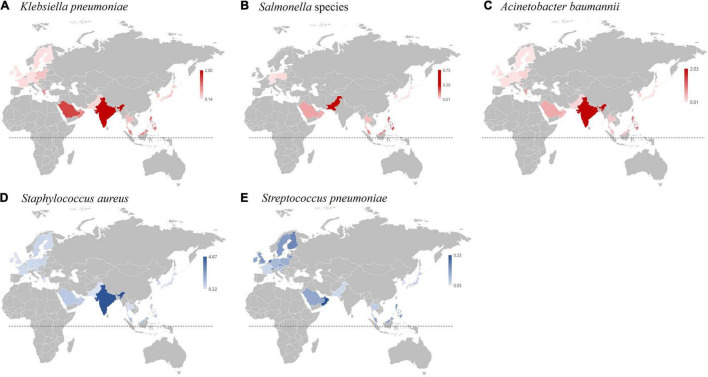
Incidence of bacterial bloodstream infection (BSI) cases relative to the incidence of *E. coli* bloodstream infection cases (rI-EC) in 2017 based on the WHO GLASS report (World Health Organization [WHO], 2019). **(A)** rI-EC of *K. pneumoniae* BSI cases. **(B)** rI-EC of *Salmonella* BSI cases. **(C)** rI-EC of *A. baumannii* BSI cases. **(D)** rI-EC of *S. aureus* BSI cases. **(E)** rI-EC of *S. pneumoniae* BSI cases. The dotted lines indicate the equatorial line.

**FIGURE 8:**
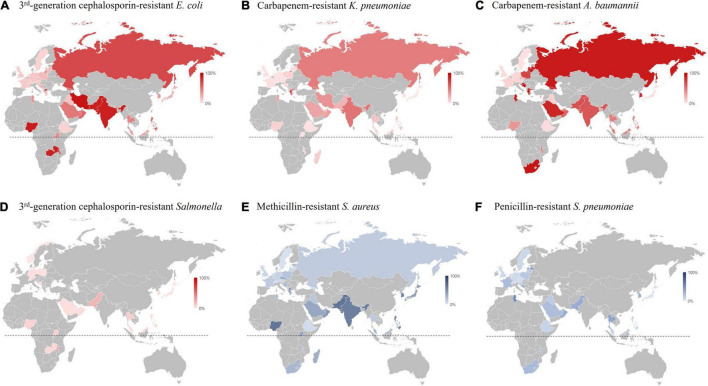
Resistance rate of major multidrug-resistant pathogens in 2017 based on the WHO GLASS report (World Health Organization [WHO], 2019). **(A)** Resistance rate to third generation cephalosporin of *E. coli* blood isolates. **(B)** Resistance rate to carbapenem of *K. pneumoniae* blood isolates. **(C)** Resistance rate to carbapenem in *A. baumannii* blood isolates. **(D)** Resistance rate to third generation cephalosporin of *Salmonella* blood isolates. **(E)** Resistance rate to methicillin in *S. aureus* blood isolates. **(F)** Resistance rate to penicillin in *S. pneumoniae* blood isolates. The dotted lines indicate the equatorial line.

The rI-Ec of the *K. pneumonia*-BSI in 2017 in South Korea was 0.35 (783/2,219), and the value was in the middle of those in countries participating in 2017 GLASS (World Health Organization [WHO], 2019). The geographic location of the country was likely associated with the rI-Ecs, i.e., high in the middle- and low-latitude countries, such as India (2.00), Saudi Arabia (1.43), Greece (0.93), and Malaysia (0.92), and low in the Northern European countries, such as Finland (0.14), Ireland (0.15), and Sweden (0.18) (Figure 7). In addition, the different rI-Ecs were also associated with the AMR in the selected indicator, as the former three countries exhibited high rates (>50%) of carbapenem resistance in *K. pneumoniae* blood isolates (Sekar et al., 2016; European Centre for Disease Prevention and Control [ECDC], 2020), and the latter three countries had low rates (<5%) of carbapenem resistance (World Health Organization [WHO], 2019). Similarly, the rI-Ecs of *A. baumannii*-BSI were high in India (2.03), the Philippines (1.00), Greece (0.74), and Saudi Arabia (0.53), with a high (>90%) rate of carbapenem resistance in *A. baumannii*, and low in Austria (0.01), Finland (0.01), Sweden (0.01), and Germany (0.02), with low (<10%) rates of carbapenem resistance (Figure 8). Notably, the rI-Ec of *A. baumannii*-BSI was 0.12 (257/2,219) in South Korea, whose rate of carbapenem resistance was 90.5%.

The rI-Ec of the *Salmonella*-BSI was very low in South Korea (0.03, 68/2,219), and it was likely associated with the socioeconomic status of each country; for example, it was high in Pakistan (0.75) and the Philippines (0.25). Notably, the major food- and waterborne pathogen *Salmonella* is a public burden in low- to middle-income countries in Southeast Asia in the tropical climatic zone (Crump et al., 2015). The rI-Ecs of *S. pneumoniae*-BSI were higher in European countries, including the Netherlands (0.21), Finland (0.16), Switzerland (0.13), the United Kingdom (0.13), and Sweden (0.13), than in South Korea (0.03, 66/2,219). Notably, *S. pneumoniae* easily colonizes dry-cold zone residences, and the incidence of invasive pneumococcal infection in the Scandinavian countries is high (Dowell et al., 2003).

The incidence of *E. faecium*-BSI was 1.4 times as high as that of *E. faecalis*-BSI. Curiously, until the 2000s in South Korea, the incidence of *E. faecalis*-BSI was much higher than that of *E. faecium*-BSI (Low et al., 2001), and the reversal in proportions is likely associated with the dominant *vanA*-positive *E. faecium* strain in clinical settings. The rate of resistance to vancomycin in *E. faecium* blood isolates increased every year throughout the phase I surveillance study. A similar increase in VREfm among blood isolates has been reported in European countries such as Germany (5.9% in 2007 to 16.7% in 2016) and Switzerland (0% in 2013 to 3.9% in 2018) (Remschmidt et al., 2018; Piezzi et al., 2020). Since VREfm is an important risk factor for prolonged hospital stays and early mortality in patients with BSIs (Kim et al., 2020), close monitoring of the clonal distribution and risk factors associated with VREfm infection should be carried out.

The penicillin-resistant *E. faecalis* isolates exhibiting discrepant susceptibility phenotype to ampicillin between disk diffusion method (ampicillin-resistant) and broth microdilution method (ampicillin-susceptible) were identified through the phase I surveillance period, which needs additional concern for their association with high mortality in patients (Kim et al., 2019b). The production of β-lactamase has been reported (Ono et al., 2005), but none of them in this study produced the β-lactamase. Further investigation to reveal the mechanism for decreased susceptibility to ampicillin in ASPR *E. faecalis* isolates is needed.

Methicillin-resistant *S. aureus* is one of the most common BSI-causing gram-positive MDR pathogens. Although the prevalence of MRSA in South Korea was 49.6%, which is the highest among those in high-income countries (Supplementary Figure 6; World Health Organization [WHO], 2019), it is decreasing every year as in other countries. For instance, the population-weighted mean MRSA percentage decreased from 16.9% in 2017 to 15.5% in 2019 through the European Antimicrobial Resistance Surveillance Network (EARS-Net) (European Centre for Disease Prevention and Control [ECDC], 2018, 2020). The decrease in MRSA is probably due to the development and implementation of infection prevention and control strategies. Notably, the proportion of MRSA diminished more in HO infection than in CO infection in this study. The SCC*mec* type II MRSA of the New York/Japan clone, notorious for its high mortality in patients with BSIs (Kim et al., 2019a), decreased every year, and the clone was replaced by the USA300. The USA300 clone, which is the common community-associated MRSA in the United States (Diekema et al., 2014), disseminated in Europe and Latin America in the late 2000s (Larsen et al., 2007; Reyes et al., 2009), as well as in Japan (Takadama et al., 2018).

*Escherichia coli* exhibited higher resistance rates to cefotaxime than to ceftazidime, probably due to the dissemination of *bla*_CTX–M–14_-positive isolates, which exhibit strong hydrolytic activity to cefotaxime but not to ceftazidime (Park et al., 2014). It was noteworthy that *bla*_CTX–M–27_-positive *E. coli* blood isolates increased every year. CTX-M-27 exhibited expanded hydrolytic activity to ceftazidime with the substitution CTX-M-14_D240G_ (Bonnet et al., 2003). The spread of *bla*_CTX–M–27_-positivie *E. coli* has been reported worldwide, including in Japan, China, Germany, France, Portugal, and Germany (Bevan et al., 2017), and the clonal spread of *E. coli* ST131 clade 1/H30R is responsible for this dissemination (Matsumura et al., 2015). In *K. pneumoniae*, both CTX-M-15 and CTX-M-55 belonging to Group 1 CTX-M ESBL were predominant and have hydrolytic activity not only to cefotaxime but also to ceftazidime. Carbapenemase gene-harboring Enterobacterales were still rare among the blood isolates, and only nine *E. coli* isolates possessing either *bla*_KPC_-, *bla*_NDM–5_, or *bla*_OXA–181_, and 23 *bla*_KPC_-positive *K. pneumoniae* isolates were identified.

The most common acquired carbapenemase gene in *P. aeruginosa* blood isolates was *bla*_IMP–6_, followed by *bla*_NDM–1_ and *bla*_GES_, while the *bla*_VIM–2_ gene is the dominant genotype in most countries, including Greece, Portugal, Italy, and Canada (Castanheira et al., 2014; Botelho et al., 2018; Giani et al., 2018; Yoon and Jeong, 2021). The ST235 *P. aeruginosa* isolates harboring *bla*_IMP–6_ has been a predominant clone in South Korea (Yoon and Jeong, 2021). The carbapenemase genes in *P. aeruginosa* are often found in the genomic islands PAGI-15 and PAGI-16 (Hong et al., 2016) and are closely associated with the amikacin resistance conferred by the aminoglycoside modifying enzyme-encoding *aacA* genes co-carried by the resistance genomic islands (Ballaben et al., 2018).

The high rate of resistance to carbapenems in *A. baumannii* blood isolates was responsible for the dominant *bla*_OXA–23_-harboring isolates. The dominant IS*Aba1-bla*_OXA–51_–carrying carbapenem-resistant *A. baumannii* in the mid-2000s in South Korea (Lee et al., 2009) was replaced completely by *bla*_OXA–23_ gene-carrying isolates. Similarly, in Greece and Italy, the endemic OXA-58-producing *A. baumannii* was replaced by OXA-23 producers (D’Arezzo et al., 2011; Karampatakis et al., 2017). Furthermore, the chromosomal integration and multiplication of *bla*_OXA–23_-associated transposons was reported in South Korea, which also contributed to the dissemination of this clone (Yoon et al., 2017).

The representativeness of the limited sentinel hospitals is always under debate for national AMR surveillance studies, and Kor-GLASS is not an exception. Unlimited increases in the number of participating sentinel hospitals are not the solution, and WHO, the core body of GLASS, takes into account the appropriate number of participating hospitals to represent the population. Two more sentinel hospitals have been included in phase I of Kor-GLASS since 2017 to cover two further districts; however, consideration of the proper sample number is still needed. Finally, molecular epidemiology is always desirable. For a better alarm system for newly emerging novel AMR, molecular characterization is a necessary step, and infrastructure for characterization should be established.

Through the operation of phase I Kor-GLASS, comprehensive AMR surveillance data were provided, and the defined molecular mechanisms of resistance helped us to better understand AMR epidemiology. AMR genotyping and strain typing enabled us to determine the clonal distribution and exchanges of major MDR clonal groups. Modifications in GLASS methodology, including the addition of specimen types, target pathogens, and molecular characterization of AMR determinants, are needed to consolidate the global surveillance of AMR, and in-depth international comparison by the strengthened GLASS system is expected in the near future.

## Data Availability Statement

The original contributions presented in the study are included in the article/Supplementary Material, further inquiries can be directed to the corresponding author.

## Ethics Statement

The studies involving human participants were reviewed and approved by the Yonsei University College of Medicine, Gangnam Severance Hospital. Written informed consent from the participants’ legal guardian/next of kin was not required to participate in this study in accordance with the national legislation and the institutional requirements.

## Author Contributions

SJ: conceiving the surveillance project. SJ, JK, and SL: managing the study. DK, E-JY, JH, MC, and SJ: operating the surveillance system. DK and SJ: analyzing the data. DK, E-JY, and SJ: writing the manuscript. JH, HK, YRK, YAK, YU, KS, JeS, EW, SK, and JoS: collecting the bacterial strains and clinical data. JP and KP: operating the quality control center. JH, MC, HK, YRK, YAK, YU, KS, JeS, JP, KP, EW, SK, JoS, and JK: critically reading the manuscript. All authors contributed to the article and approved the submitted version.

## Conflict of Interest

The authors declare that the research was conducted in the absence of any commercial or financial relationships that could be construed as a potential conflict of interest.

## Publisher’s Note

All claims expressed in this article are solely those of the authors and do not necessarily represent those of their affiliated organizations, or those of the publisher, the editors and the reviewers. Any product that may be evaluated in this article, or claim that may be made by its manufacturer, is not guaranteed or endorsed by the publisher.
