# Supplementary material for: Major Bloodstream Infection-Causing Bacterial Pathogens and Their Antimicrobial Resistance in South Korea, 2017–2019: Phase I Report From Kor-GLASS
Source: Front Microbiol. 2022 Jan 6;12:799084. doi: 10.3389/fmicb.2021.799084 (PMC8770956; doi:10.3389/fmicb.2021.799084)
Supplement: Supplementary file 1 [file Data_Sheet_1.docx]

Table S1. The antimicrobials tested for each bacterial species in this study

| Bacterial species | Antimicrobial susceptibility testing method | Antimicrobial agents | Quality control strain |
| --- | --- | --- | --- |
| *S. aureus* | Disk diffusion | Cefoxitin, erythromycin, clindamycin, quinupristin-dalfopristin, trimethoprim-sulfamethoxazole, mupirocin, vancomycin, teicoplanin, linezolid, tigecycline | ATCC 25923 (*S. aureus*) |
|  | Broth microdilution | Vancomycin, teicoplanin, linezolid, tigecycline | ATCC 29213 (*S. aureus*) |
| Enterococci | Disk diffusion | Ampicillin, penicillin, ciprofloxacin, gentamicin, streptomycin, tetracycline | ATCC 29212 (*E. faecalis*) |
|  | Broth microdilution | Vancomycin, teicoplanin, linezolid, tigecycline | ATCC 29212 (*E. faecalis*) |
| *S. pneumoniae* | Broth microdilution | Penicillin, amoxacillin-clavulanate, cefotaxime, ceftriaxone, cefuroxime, erythromycin, levofloxacin, trimethoprim-sulfamethoxazole | ATCC 49619 (*S. pneumoniae*) |
| *E. coli* | Disk diffusion | Ampicillin, piperacillin, ampicillin-sulbactam, cefazolin, cefotaxime, ceftazidime, cefepime, aztreonam, cefoxitin, imipenem, meropenem, ertapenem, amikacin, gentamicin, ciprofloxacin, trimethoprim-sulfamethoxazole, tigecycline | ATCC 25922 (*E. coli*) |
|  | Broth microdilution | Colistin | ATCC 25922 (*E. coli*)  ATCC 27853 (*P. aeruginosa*) |
| *K. pneumoniae* | Disk diffusion | Piperacillin, ampicillin-sulbactam, cefazolin, cefotaxime, ceftazidime, cefepime, aztreonam, cefoxitin, imipenem, meropenem, ertapenem, amikacin, gentamicin, ciprofloxacin, trimethoprim-sulfamethoxazole, tigecycline | ATCC 25922 (*E. coli*) |
|  | Broth microdilution | Colistin | ATCC 25922 (*E. coli*)  ATCC 27853 (*P. aeruginosa*) |
| *Salmonella* spp. | Broth microdilution | Azithromycin, cefotaxime, ceftazidime, ciprofloxacin, imipenem | ATCC 25922 (*E. coli*) |
| *P. aeruginosa* | Disk diffusion | Piperacillin, piperacillin-tazabactam, ceftazidime, cefepime, imipenem, meropenem, amikacin, gentamicin, tobramycin, ciprofloxacin | ATCC 25922 (*E. coli*)  ATCC 27853 (*P. aeruginosa*) |
|  | Broth microdilution | Colistin | ATCC 25922 (*E. coli*)  ATCC 27853 (*P. aeruginosa*) |
| *Acinetobacter* spp. | Disk diffusion | Piperacillin, ampicillin-sulbactam, ceftazidime, cefepime, imipenem, meropenem, amikacin, gentamicin, tobramycin, ciprofloxacin, minocycline, tigecycline | ATCC 25922 (*E. coli*)  ATCC 27853 (*P. aeruginosa*) |
|  | Broth microdilution | Colistin | ATCC 25922 (*E. coli*)  ATCC 27853 (*P. aeruginosa*) |

Table S3. Distribution of the *spa* types of the methicillin-resistant *S. aureus* isolates stratified according to the SCC*mec* type

| ***spa* type** | **Total MRSA isolates**  **(n = 1059)** |  | **SCC*mec* type II MRSA**  **(n = 503)** | **SCC*mec* type IV MRSA**  **(n = 531)** | **SCC*mec* type V MRSA**  **(n = 10)** | **Other SCC*mec* types**  **(n = 15)** |
| --- | --- | --- | --- | --- | --- | --- |
| t2460 | 217 (20.5) |  | 216 (42.9) | 0 (0) | 0 (0) | 1 (6.7) |
| t324 | 140 (13.2) |  | 3 (0.6) | 135 (25.4) | 0 (0) | 2 (13.3) |
| t008 | 94 (8.9) |  | 1 (0.2) | 92 (17.3) | 0 (0) | 1 (6.7) |
| t148 | 76 (7.2) |  | 3 (0.6) | 73 (13.7) | 0 (0) | 0 (0) |
| t002 | 64 (6.0) |  | 62 (12.3) | 2 (0.4) | 0 (0) | 0 (0) |
| t664 | 52 (4.9) |  | 4 (0.8) | 48 (9.0) | 0 (0) | 0 (0) |
| t111 | 51 (4.8) |  | 51 (10.1) | 0 (0) | 0 (0) | 0 (0) |
| t9353 | 33 (3.1) |  | 32 (6.4) | 1 (0.2) | 0 (0) | 0 (0) |
| t2431 | 23 (2.2) |  | 2 (0.4) | 21 (4.0) | 0 (0) | 0 (0) |
| t189 | 19 (1.8) |  | 1 (0.2) | 14 (2.6) | 4 (40.0) | 0 (0) |
| t2461 | 13 (1.2) |  | 0 (0) | 13 (2.4) | 0 (0) | 0 (0) |
| t1784 | 11 (1.0) |  | 1 (0.2) | 10 (1.9) | 0 (0) | 0 (0) |
| t1767 | 10 (0.9) |  | 1 (0.2) | 9 (1.7) | 0 (0) | 0 (0) |
| t264 | 10 (0.9) |  | 9 (1.8) | 0 (0) | 0 (0) | 1 (6.7) |
| t3092 | 5 (0.5) |  | 0 (0) | 3 (0.6) | 1 (10.0) | 1 (6.7) |
| t034 | 2 (0.5) |  | 0 (0) | 0 (0) | 2 (20.0) | 0 (0) |
| t571 | 2 (0.2) |  | 0 (0) | 0 (0) | 1 (10.0) | 1 (6.7) |
| t311 | 1 (0.1) |  | 0 (0) | 0 (0) | 1 (10.0) | 0 (0) |
| t5554 | 1 (0.1) |  | 0 (0) | 0 (0) | 1 (10.0) | 0 (0) |
| Others | 235 (22.2) |  | 117 (23.3) | 110 (20.7) | 0 (0) | 8 (53.3) |


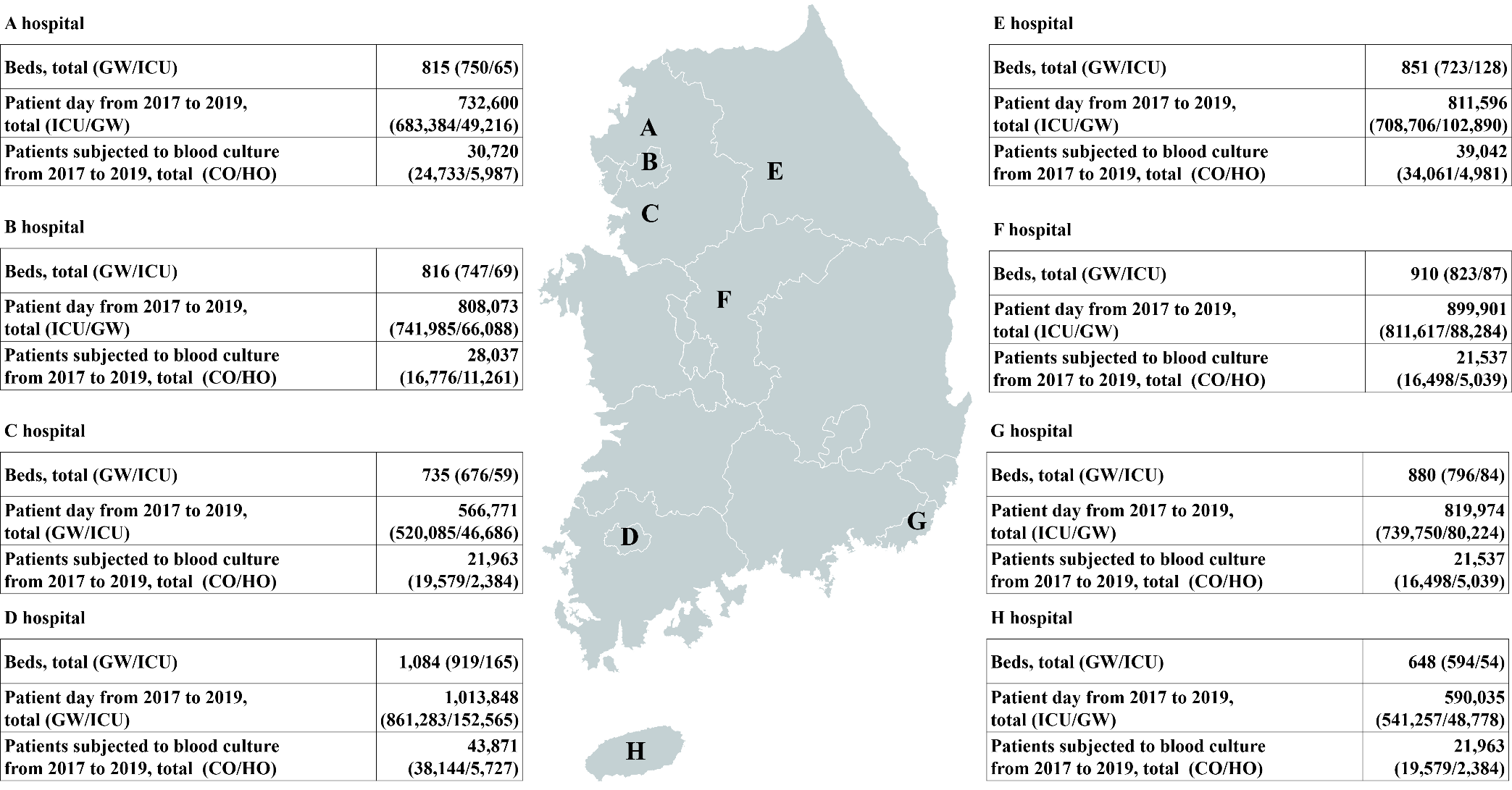


Figure S1. Hospital size in beds, number of patient-days, and number of patients with suspected bloodstream infection by hospital during the study period.


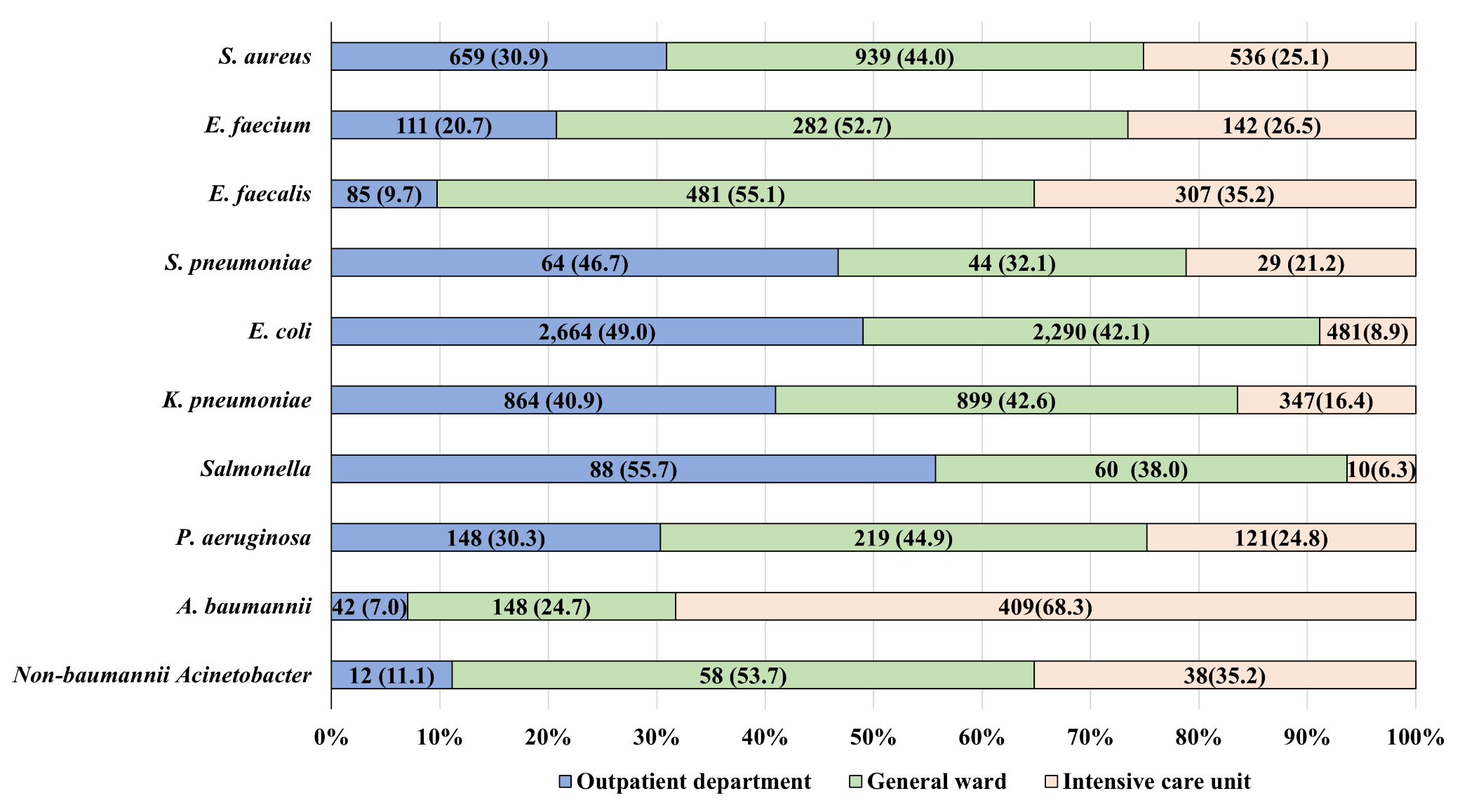


Figure S2. Occurrence of bloodstream infection cases by admission type.


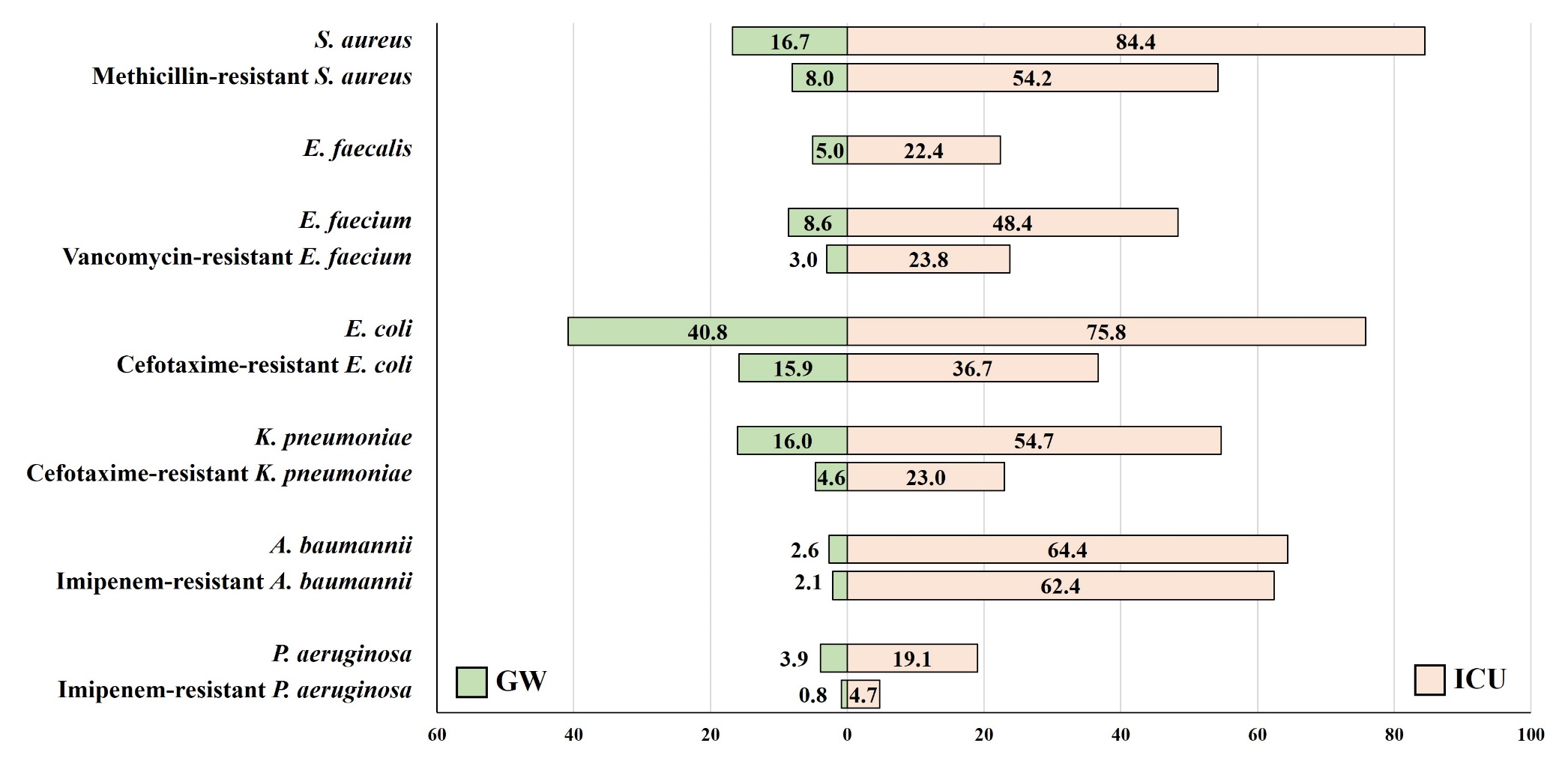


Figure S3. Occurrence of bloodstream infections per 100,000 patient-days by pathogen.


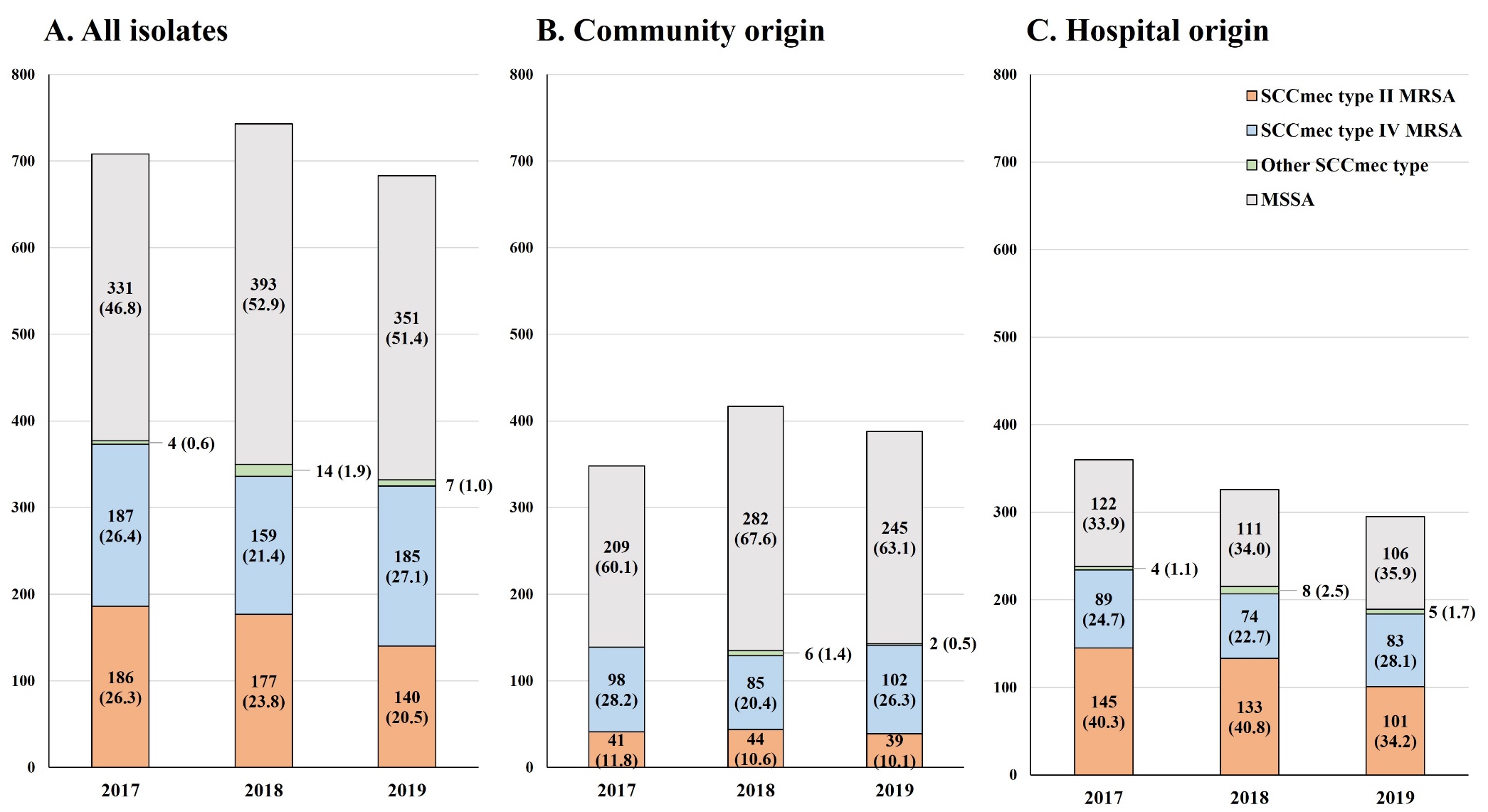


Figure S4. The staphylococcal cassette *mec* type of methicillin-resistant *S. aureus* by type of infection.


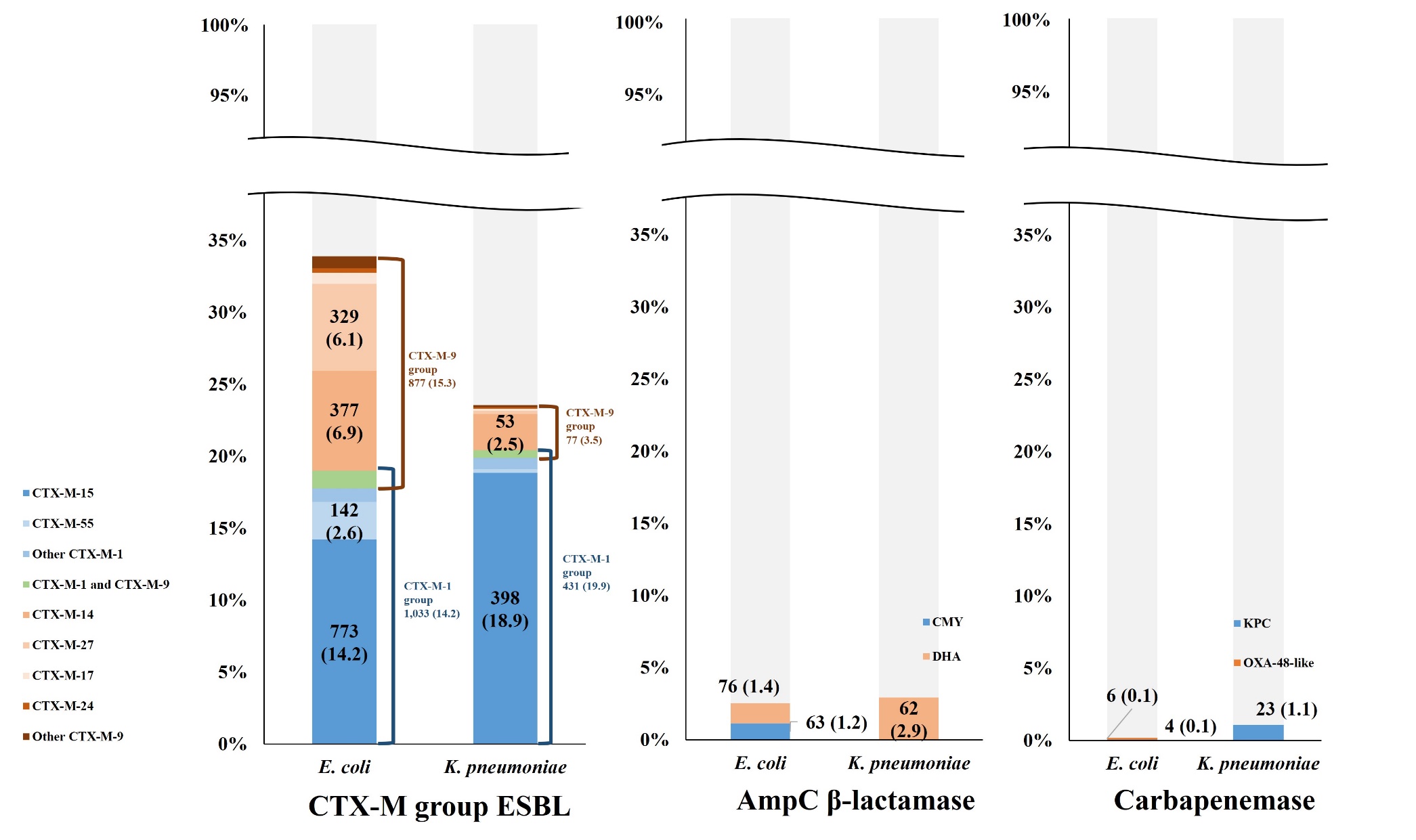
Figure S5. Distribution of beta-lactamases in *E. coli* and *K. pneumoniae* blood isolates.


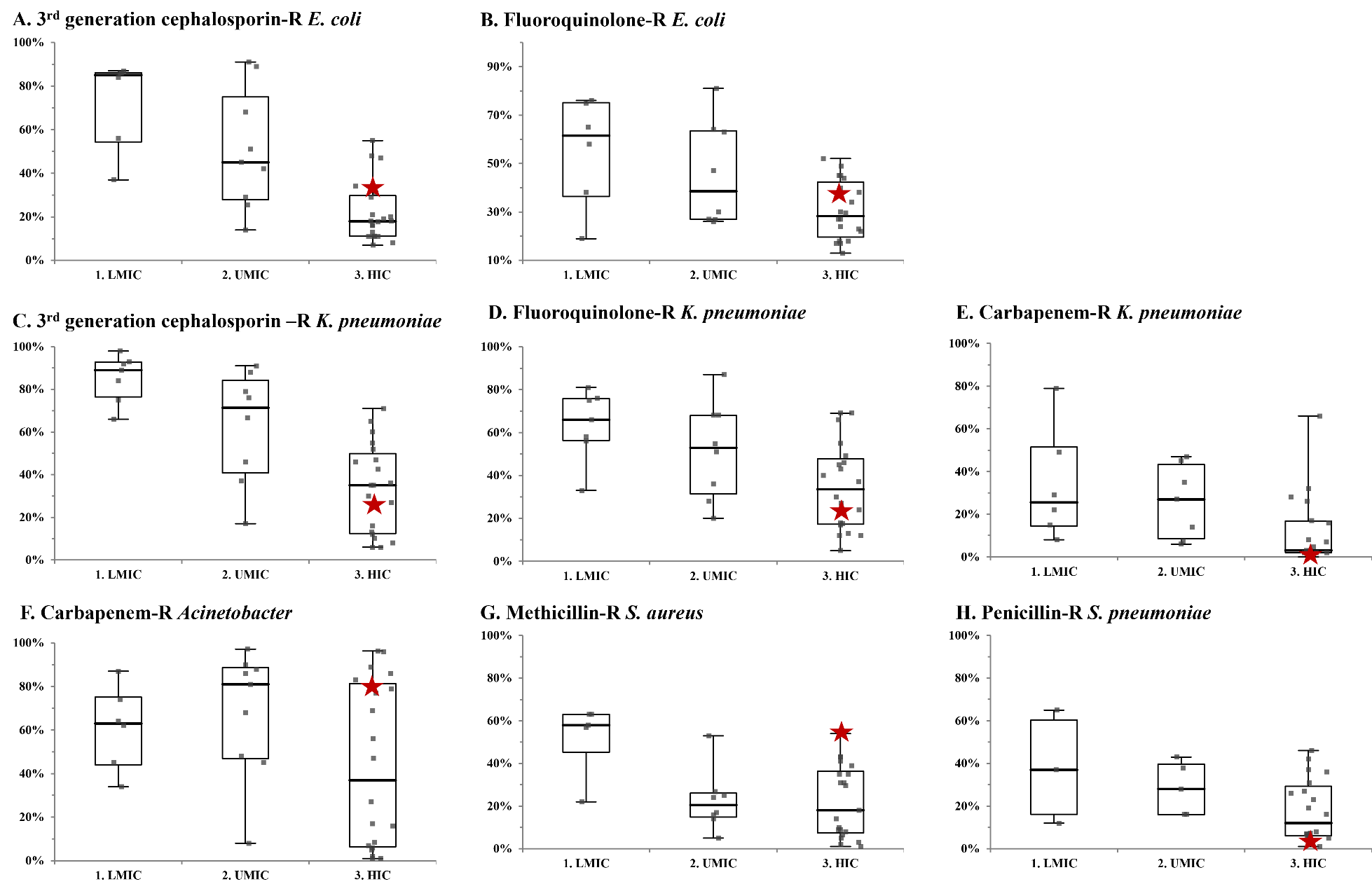


Figure S6. Percentages of resistance to major antimicrobials by socioeconomic status according to GLASS report 2017 [13]. The red stars indicate the resistance rate in South Korea.
